# Adult neurogenesis and specific replacement of interneuron subtypes in the mouse main olfactory bulb

**DOI:** 10.1186/1471-2202-8-92

**Published:** 2007-11-09

**Authors:** Joshua Bagley, Greg LaRocca, Daniel A Jimenez, Nathaniel N Urban

**Affiliations:** 1 Department of Biological Science, Carnegie Mellon University, Pittsburgh, PA 15213, USA; 2 Department of Neuroscience University of Pittsburgh, Pittsburgh, PA 15260, USA; 3 Center for the Neural Basis of Cognition, Pittsburgh, PA 15213, USA; 4 Center for Neuroscience at the University of Pittsburgh, Pittsburgh, PA 15213, USA; 5 National Institutes of Health/NINDS, 35 Convent Drive, Bldg.35/Rm. #A108, Bethesda, MD 20892-3703, USA

## Abstract

**Background:**

New neurons are generated in the adult brain from stem cells found in the subventricular zone (SVZ). These cells proliferate in the SVZ, generating neuroblasts which then migrate to the main olfactory bulb (MOB), ending their migration in the glomerular layer (GLL) and the granule cell layer (GCL) of the MOB. Neuronal populations in these layers undergo turnover throughout life, but whether all neuronal subtypes found in these areas are replaced and when neurons begin to express subtype-specific markers is not known.

**Results:**

Here we use BrdU injections and immunohistochemistry against (calretinin, calbindin, N-copein, tyrosine hydroxylase and GABA) and show that adult-generated neurons express markers of all major subtypes of neurons in the GLL and GCL. Moreover, the fractions of new neurons that express subtype-specific markers at 40 and 75 days post BrdU injection are very similar to the fractions of all neurons expressing these markers. We also show that many neurons in the glomerular layer do not express NeuN, but are readily and specifically labeled by the fluorescent nissl stain Neurotrace.

**Conclusion:**

The expression of neuronal subtype-specific markers by new neurons in the GLL and GCL changes rapidly during the period from 14–40 days after BrdU injection before reaching adult levels. This period may represent a critical window for cell fate specification similar to that observed for neuronal survival.

## Background

In adult rodents, neuronal stem cells (NSCs) divide in the subventricular zone (SVZ), and then migrate tangentially as neuroblasts along the rostral migratory stream (RMS) to the main olfactory bulb (MOB). Upon reaching the olfactory bulb, these cells migrate radially throughout the laminar structure of the MOB eventually coming to rest in the granule cell layer (GCL) and glomerular layer (GLL) [1-4]. In rodents, neurogenesis results in ongoing addition of cells to these two layers throughout life. Simultaneously neurons in these same areas undergo apoptosis, suggesting that existing neurons in the bulb are replaced by the newly generated neurons [3,5-7]. Once in the MOB, approximately 50–70% of the new neurons within the GCL die by day 40–45 after neuronal birth [8,9]. After this period the number of new neurons decreases much more slowly, suggesting that a fraction of the initial population of new neurons integrates in a relatively permanent fashion into the functional circuitry [5,8,10]. This functional integration involves developing adult-like morphology [4,8] and physiology [4,8,11,12] and presumably connectivity.

Here we describe another aspect of the maturation of neuroblasts into adult neurons: the differentiation of newly generated neurons into immunohistochemically-defined neuronal subtypes. The GCL consists primarily of granule cells, although other cell types have been described [13,14]. The GLL contains an even greater diversity of cell types that are distinguished by morphological [15], physiological [16] and immunohistochemical [17-20] properties. Neurons in both the GLL and GCL express a diversity of immunohistochemically defined biochemical subtypes such as calbindin (CB), calretinin (CR), N-copine (NC), parvalbumin (PV), and GABA [17-22]. These markers are expressed by many cells in these layers. In some cells the markers are individually expressed, and in other cells a combination of various markers may be expressed [17]. The functional role of different interneuron subtypes is not clear, but expression of these markers is differentially regulated by activity [23,24] and associated differences in morphology and connectivity. Moreover physiological differences in periglomerular cells have been observed [16] although very limited information is available on whether there is any correlation between physiological classes and immunohistochemical markers in the olfactory bulb. The expression of many of these markers by adult-born neurons has been recently characterized indicating that several major classes of PG cells are generated from subventricular zone precursors [25,26].

BrdU labeling of dividing cells shows that the vast majority of adult-born cells become neurons and that these neurons begin to appear in the MOB within a week after their last division [6,8]. Between 14 and 28 days after BrdU injection the number of BrdU positive cells in the MOB decreases substantially, and this decrease can be regulated by changes in neuronal activity [8,9]. Here we examine the specification of neuronal phenotype in new neurons and report that adult-born neurons gradually develop adult distributions of immunohistochemical markers in the period between 14–40 days after BrdU injection, suggesting that there may be a critical period during which neuronal subtype is specified.

## Results

### BrdU immunohistochemistry

**Figure 1:**
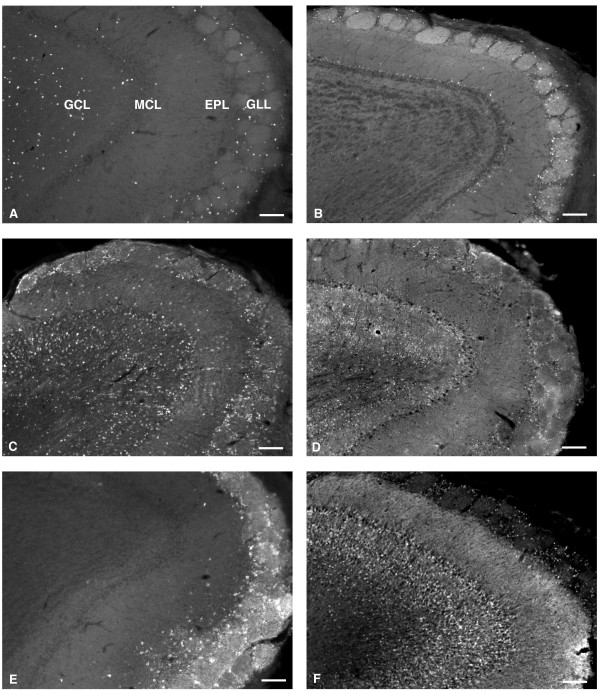
BrdU-positive cells and also cells staining for the biochemical subtype markers CB, CR, GABA, TH, and NC are differentially distributed throughout the olfactory bulb. Low magnification images (10×) of sagittal sections through the olfactory bulb show positive immunoreactivity for (A) BrdU, (B) CB, (C) CR, (D) GABA, (E) TH, and (F) NC predominately within the GLL and GCL. Scale bars, 100 μm. CB, calbindin; CR, calretinin; TH, tyrosine-hydroxylase; NC, N-copine; GLL, glomerular layer; GCL, granule cell layer.

Using BrdU as a marker for adult generated neurons, we first examined the final destination of the migrating new neurons within the olfactory bulb at 40 days after BrdU injection. BrdU positive nuclei had a circular morphology, not elongated as seen for migrating cells. As reported previously [1,8], BrdU positive cells were seen in both GLL and GCL layers (Fig. 1A) with the majority of positive labeling occurring in the GCL. Within the GLL a smaller number of BrdU positive cells were observed whereas little or no BrdU staining was observed in the mitral cell layer (Fig. 1A).

### CB, CR, GABA, NC, and TH immunohistochemistry

We next stained olfactory bulb sections with antibodies against a panel of marker proteins that have previously been shown to have unique expression patterns within the GLL and GCL of the MOB. Olfactory bulb sections (see methods) from mice (see additional file 1) sacrificed 40 days after final BrdU injection were examined to determine the expression patterns of calbindin (CB), calretinin (CR), GABA, N-copine (NC), and tyrosine hydroxylase (TH) (Figure 1B–F). The labeling we observed for these markers was in agreement with previous studies [17-22]: CB labeling was perisomatic and primarily observed in the GLL region with few GCL and MCL positive cells (Fig. 1B). CR labeling was perisomatic and seen in GLL and mostly in the superficial GCL (Fig. 1C). Dense GABA labeling was cytoplasmic and seen throughout the GLL and GCL (Fig. 1D). TH labeling was perisomatic and punctate in dendrites, and observed principally in the GLL with very few GCL positive cells (Fig. 1E). For this reason, in subsequent experiments, counting of TH positive cells in the GCL was not performed. NC labeling was cytoplasmic with some labeling of proximal dendrites, and occurred primarily in the GCL with less intense and sparse labeling in the GLL (Fig. 1F).

### New neurons express markers of several different neuronal subtypes

**Figure 2:**
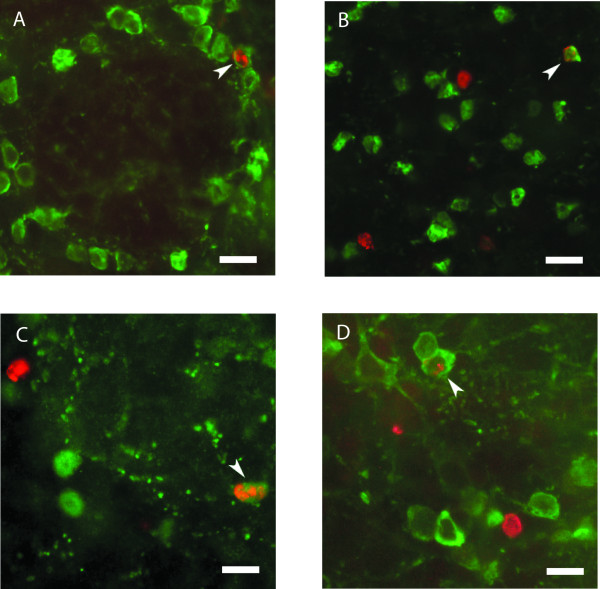
Adult born neurons express CR, CB, and TH. 40× images with arrowheads indicating double positive cells for BrdU (red) and the subtypes CR, CB, and TH (green). Scale bars, 37 μm. (A and B) BrdU co-labels with CR in the GLL (A) and the GCL (B). BrdU co-labels with CB (C) and TH (D) in the GLL. CB, calbindin; CR, calretinin; TH, tyrosine-hydroxylase; GLL, glomerular layer; GCL, granule cell layer.

**Figure 3:**
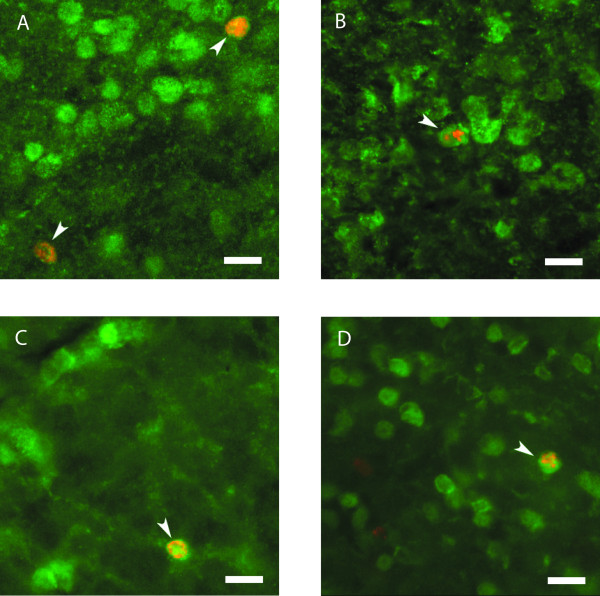
Adult born neurons express GABA and NC. 40× images of sagittal sections with arrowheads indicating double positive cells for BrdU (red) and the subtypes GABA and NC (green). Scale bars, 37 μm. (A and B) BrdU co-labels with GABA in the GLL (A) and the GCL (B). (C and D) BrdU co-labels with NC in the GCL (C) and TH (D) in the GLL. NC, N-copine; GLL, glomerular layer; GCL, granule cell layer.

**Figure 4:**
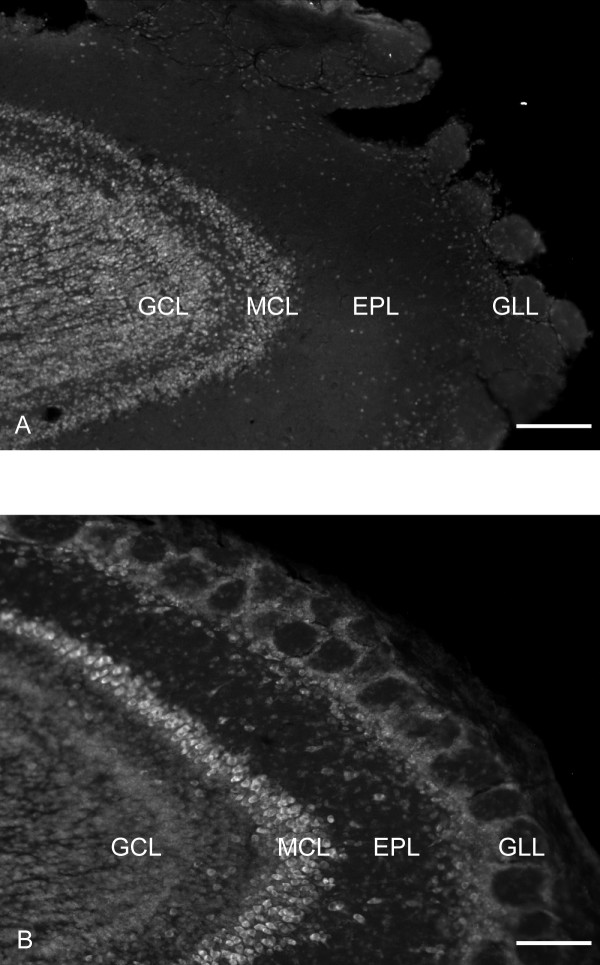
The neuronal specific marker NeuN does not label all GLL neurons. 10× images of sagittal sections shown to compare the immunofluorescent staining of NeuN (A) to that of the fluorescent Nissl stain NT (B) within the olfactory bulb. NT stains a much larger number of cells in the GLL. Scale bars, 148 μm. NeuN, neuronal nuclei; GLL, glomerular layer; NT, NeuroTrace; GCL, granule cell layer; MCL, mitral cell layer; EPL, external plexiform layer.

The expression patterns of each of the subtype markers are distinctive while new neurons observed 40 days after BrdU injection reside fairly uniformly throughout the GLL and GCL. Thus, we tested whether the new neurons may represent a variety of immunohistochemically-defined neuronal subtypes or whether a few subtypes are selectively replaced by new neurons. To assess this quantitatively, we determined the fraction of BrdU-positive cells that co-expressed each of the immunohistochemical markers described above. Adult mice (7–9 weeks old, additional file 1) were injected with BrdU and then perfused 40 days after the final BrdU injection. Sections were stained for BrdU and one other marker (see table 1). We identified BrdU positive cells in the GLL and then determined the number and percentage of these cells that were also positive for CR (28 of 123 cells; 23 ± 2%), CB (22 of 169 cells; 14 ± 3%), TH (34 of 149 cells; 23 ± 4%), GABA (129 of 215 cells; 60 ± 2%), and NC (11 of 99 cells; 12 ± 2% (Fig. 2, 3 and 4). The ssame analysis was performed on the BrdU positive neurons in the GCL (Fig. 2, 3 and 4), except that, as stated above, TH-positive cells were not analyzed. The numbers and percentages of GCL neurons expressing the different markers are as follows: CR (302 of 3502 cells; 9 ± 1%), CB (69 of 4127 cells; 2 ± 0%), GABA (319 of 504 cells; 62 ± 6%), and NC (50 of 229 cells; 22 ± 2%).

**Table 1:** Antibodies, Dilutions, Sources

Primary Antibody	Source	Dilution	Secondary Antibody (Molecular Probes)	Dilution
Rat anti-BrdU	Serotec, MCA2060	1:1000	Donkey anti-rat AlexaFluor 594	1:600
Rabbit anti-Calbindin (CB)	Chemicon, AB1778	1:1000	Donkey anti-rabbit AlexaFluor 488	1:600
Goat anti-Calretinin (CR)	Santa Cruz, sc-11644	1:1500	Donkey anti-goat AlexaFluor 488	1:600
Mouse anti-GABA	Sigma, A0310	1:2000	Donkey anti-goat AlexaFluor 488	1:600
Mouse anti-N-copine (NC)	Transduction Labs C10220	1:2000	Donkey anti-mouse AlexaFluor 488	1:600
Sheep anti-Tyrosine hydroxylase (TH)	Calbiochem, 657014	1:1000	Donkey anti-sheep AlexaFluor 488	1:600
Rabbit anti-S100β	Swant, 37-A	1:5000	Donkey anti-rabbit AlexaFluor 488	1:600
Mouse anti-neuronal nuclei (NeuN)	Chemicon, MAB377	1:1000	Donkey anti-mouse AlexaFluor 488 or 594	1:600

These data indicate that the new neurons in both the GLL and GCL express several of the major immunohistochemical markers previously found in the cells of these layers. We next wanted to compare the fraction of BrdU positive cells expressing these markers to the fraction of all neurons in these layers expressing these markers. We evaluated two neuron-specific markers for this purpose.

### NeuN does not label all neurons in the GLL

**Figure 5:**
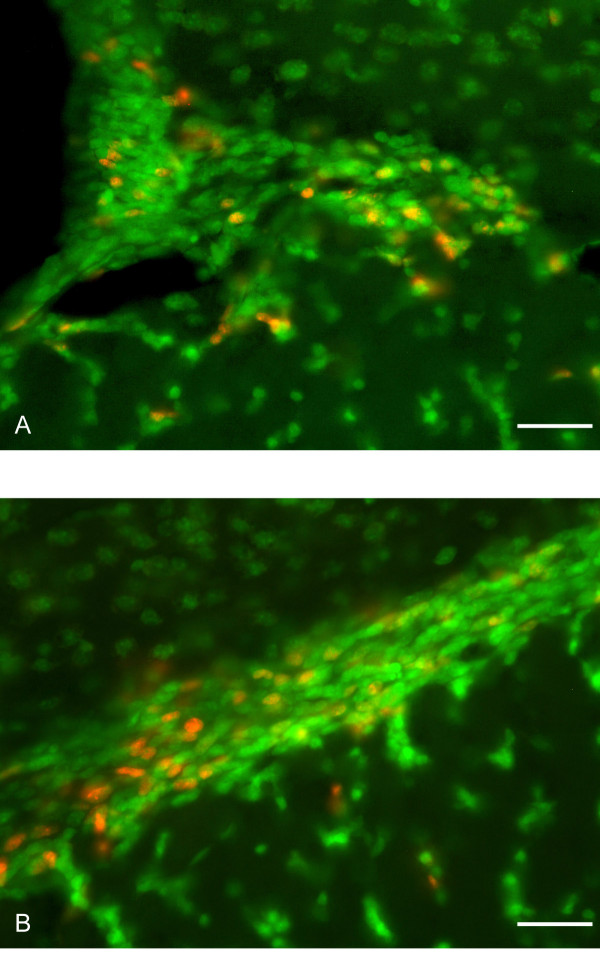
Adult born neurons express the neuron specific marker NT in the SVZ and proximal RMS 12 hours after the final BrdU injection. 40× images of sagittal sections through the olfactory bulb of SVZ showing BrdU (red) and NT (green). Co-localization is seen within the SVZ (A) and the proximal RMS (B). Scale bars, 37 μm. NT, NeuroTrace; SVZ, sub-ventricular zone; RMS, rostral migratory stream, BrdU, bromodeoxyuridine.

NeuN is a marker of adult neurons that has been used previously to identify BrdU positive cells as neurons (as opposed to glia) [27]. However, certain cell types, notably cerebellar Purkinje cells and olfactory bulb mitral cells do not consistently stain for NeuN [27]. In initial studies, we observed that relatively few GLL cells seemed to express NeuN, consistent with a recent report [28]. Thus we compared the staining of NeuN and another potentially neuron-specific marker, the fluorescent Nissl stain Neuro Trace (NT). NT and NeuN both showed the same robust labeling in the GCL, but in the GLL many cells that were labeled by NT were not stained for NeuN, indicating that NeuN does not label all PG neurons (Fig. 5). Of the cells stained by NT, greater than 95% in the GCL and only 15% in the GLL also express NeuN. In contrast, all NeuN positive cells express NT. Based on these observations we further tested NT as a candidate label for the total population of neurons within the GCL and GLL of the MOB. Sections were stained with NT and for the glial marker S-100B to determine how specific NT staining was for neurons vs glia. We found that only about 2% (14/640) of NT labeled cells in the glomerular layer were also positive for S-100B. This is comparable to the fraction of NeuN positive cells that were identified as being S-100B positive in this same layer in tissue from the same animals (4/640). Similar fractional overlap was found for the granule cell layer (13/640 for NT and 1/640 for NeuN).

### NT stains new and old neurons

We next analyzed the population of cells expressing NT to determine whether this represented a population of mature neurons or whether NT stained all neurons regardless of their age. Adult mice (additional file 1) (approximately 8 weeks old) were injected with BrdU to label dividing cells and then sacrificed 12 hours after the final BrdU injection, in order to examine recently divided cells early in migration (within the SVZ and RMS). Sections were stained for BrdU and NT and we examined neurons labeled with anti-BrdU to determine whether these cells also were stained by NT at this early time point. We observed most BrdU-positive cells in the area of the SVZ (Fig. 5a) and the proximal RMS (portion closest to the SVZ) (Fig. 5b). In these experiments nearly all (>90%) of the cells in both the SVZ and RMS that were BrdU positive were also stained by NT. These results indicate that NT labels neurons very early in their development; therefore all neurons within the adult MOB are likely to be labeled by NT. In addition, this supports previous observations that new neurons begin their differentiation early in their migration even as early as within the SVZ and proximal RMS [4].

### The neuronal subtypes expressed by new neurons reflect those expressed by existing MOB neurons

**Figure 6:**
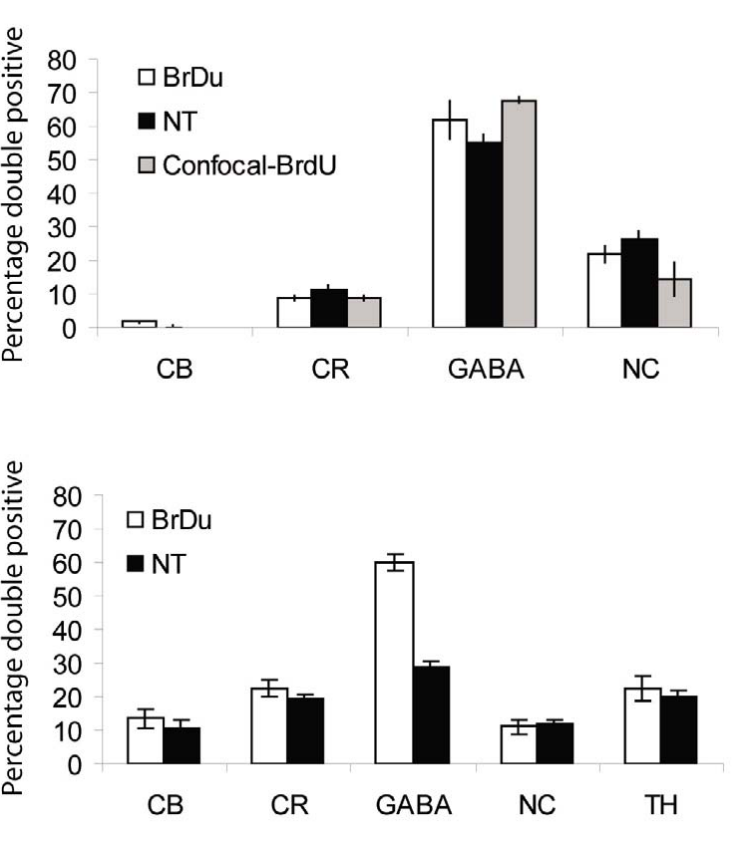
The fractions of biochemical markers expressed by adult born neurons are similar to those expressed by the overall population of olfactory bulb neurons labeled with NT. The percentage of BrdU or NT positive cells that also express NC, CR, GABA, TH, and CB in the GCL (A) and GLL (B). Data for all markers are similar except for GABA, which is expressed in significantly more new neurons (BrdU+) than in the overall population (NT+). Also shown, data from confocal imaging of new GCL neurons double labeled for Calretinin, GABA and N-copein. BrdU, bromodeoxyuridine; NT, NeuroTrace; NC, N-copine; CR, calretinin; TH, tyrosine-hydroxylase; CB, calbindin; GCL, granule cell layer; GLL, glomerular layer.

Having shown that new neurons express different immunohistochemical markers, we next asked how the rates at which new neurons express these markers compare to those of the total neuronal population in the GLL and GCL of the MOB. To examine this question, we determined the fraction of new neurons (BrdU+ cells) that also expressed the immunohistochemical markers described above and also the fraction of all neurons (NT stained neurons) that expressed these same markers. Tissue from adult mice (additional file 1, approximately 8 weeks old) sacrificed 40 days after BrdU injection was labeled with NT and each of the subtype markers (CB, CR, GABA, NC, and TH). In the GLL, the percentages of NT positive neurons that co-labeled with one of the subtypes are NC (108 of 920 cells; 12 ± 1%), CR (179 of 920 cells; 20 ± 2%), GABA (266 of 920 cells; 29 ± 2%), TH (191 of 960 cells; 20 ± 2%), and CB (100 of 957 cells; 10 ± 1%) (Fig. 6). In the GCL, the percentages of NT positive neurons co-labeled with one of the subtypes are NC (255 of 960 cells; 27 ± 2%), CR (101 of 920 cells; 11 ± 1%), GABA (516 of 930 cells; 55 ± 2%), and CB (1 of 960 cells; 0%) (Fig. 6). When compared with the percentage of BrdU cells co-expressing each subtype, the only significant difference (t test, p < .05) was observed in the GLL for GABA: A greater fraction of 40 day old neurons (60 ± 2%) expressed GABA than did NT positive neurons (29 ± 2%, p < 0.05). These results indicate that the subtypes of the new neurons at 40 days post BrdU injection are similar to that of existing neurons within the GCL and GLL, with the exception that more new neurons in the GLL also express GABA.

### New neurons show increased expression of subtype-specific markers over the period 14–40 days after their birth

**Figure 7:**
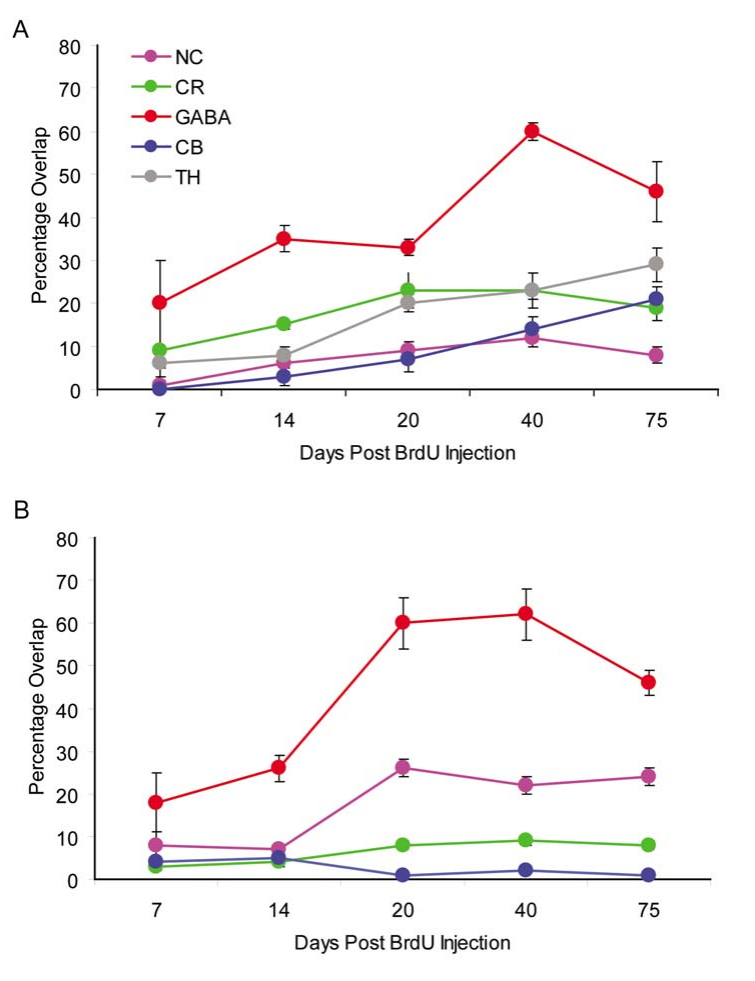
The biochemical subtype expression of adult born neurons is dynamic from 14–75 days after the final BrdU injection. The percentage of BrdU cells that co-expressed NC, CR, GABA, TH, and CB in the GCL (A) and GLL (B) at 7, 14, 20, 40 and 75 days post final BrdU injection. BrdU, bromodeoxyuridine; NC, N-copine; CR, calretinin; TH, tyrosine-hydroxylase; CB, calbindin; GCL, granule cell layer; GLL, glomerular layer.

Having examined the neuronal subtypes expressed by the new neurons after their migration/differentiation was presumed to be complete we next wanted to compare these expression rates with those of earlier time points in the differentiation process. Adult mice (approximately 8 weeks old) were injected with BrdU and groups of mice (see additional file 1) were sacrificed at 7, 14, 20, 40 and 75 days after the final BrdU injection. Sections were labeled with BrdU and one of the biochemical subtype markers (CB, CR, GABA, NC, or TH). The percentage of BrdU cells co-labeled with each subtype was determined in the GCL and GLL for each time point. The expression of all these immunohistochemical markers by the new neurons in the GCL and GLL increased almost monotonically over the period 14–40 days after BrdU injection, except for CB in the granule cell layer which showed no significant change (R^2 ^values for glomerular layer CB = 0.95, CR = 0.56, GABA = 0.66, NC = 0.60 and TH = 0.97 and for granule cell layer, CR = 0.77, GABA = 0.54, NC = 0.66, Fig. 7). The time course of expression increase was appeared slightly delayed in the GLL, relative to the GCL, but this effect did not reach significance (Fig. 7b). Staining of new neurons for GABA increased between 7 and 40 days post BrdU injection (p < 0.05) but then decreased in the GCL between days 40 and 75 (p < 0.05). Interestingly this decrease brings the fraction of new neurons staining as GABA positive closer to the fraction of all neurons that stain for GABA. These results show that the new neurons differentiate into their subtypes over the period from 14–40 days post BrdU with separate time courses within the GLL and GCL.

## Discussion

Here we have shown that adult-born neurons in the olfactory bulb differentiate into all of the major immunohistochemically-defined subtypes of granule cell layer and glomerular layer neurons examined, and the fractions of adult-born neurons differentiating into each of these immunohistochemically-defined biochemical subtypes is very similar to the fractions of neurons in the total population of each subtype. Additionally we have shown how the expression of these markers by new neurons changes over the period from the arrival of new neurons from the RMS to their incorporation into the MOB circuitry (up to 75 days).

Previously, BrdU injections into neonatal rats have shown that the fraction of BrdU-positive neurons 18 days post BrdU injection expressing GABA in the GCL (59%) and GLL (51%), and expressing TH in the GLL (10%) are similar to what we find at 20 days post injection: For GABA 60 ± 6% in GCL and 33 ± 2% in GLL, and for TH 20 ± 2% in GLL [29]. Moreover, two recent studies on the diversity of neuronal types in the mouse olfactory bulb have concluded that the fractions of neurons in the GCL and GLL that express CB, CR and TH are very similar to what we report here as being double labeled with NT [28]. One of these studies also concluded, based on staining for GAD67 and the use of GAD65-GFP mice that about 53% of GLL neurons were GABAergic and that nearly 100% of GCL neurons were GABAergic. Our data show that about 60% of GCL neurons are immunopositive for GABA. This difference may indicate that our GABA immunostaining underestimates the fraction of GABAergic neurons or that there is diversity in the amount of GABA contained in granule cells. Similarly, our data are consistent with and complementary to another recent paper by Whitman and Greer [25] which concluded that the fates assumed by adult-born virally-labeled PG neurons between 30 and 46 days post injection roughly matches the fates of the existing population of mostly mature neurons. In addition to the subtype markers we chose, this study examines the parvalbumin (PV) expressing subtype. None of these current studies examine the NC expressing subtype. However, one of the few papers on NC [22] indicates that its expression may be regulated by synaptic strength. Thus the increase in NC expression in adult-born neurons may reflect the degree to which neurons are ir integrated into the olfactory bulb circuitry rather than being a marker for a truly different subtype of neurons.

To date, clear functional roles for cells expressing these different markers have not been defined. Expression of these markers may in some cases be correlated with anatomical or physiological features of these cells, and thus they may be good markers of cell "fate". In other cases, expression of these markers may be indicators of neuronal activity or other transient neuronal features. TH-positive neurons are an interesting example of these two interpretations of these staining results. TH-positive neurons are believed to arise from a specialized precursor population, and yet TH expression levels are also activity-dependent [30,31]. Since our animals were housed under very similar conditions, it is most likely that the major factor in regulating the expression of these markers are the normal processes of cellular development.

These results suggest that the differentiation of olfactory bulb interneurons into subtypes is regulated in a way that is uniform across postnatal development. This conclusion is somewhat at odds with data in which cells harvested from embryonic, neonatal or adult mice were transplanted into the SVZ of neonatal and adult recipients [26]. This study concluded that different fractions of transplanted neurons integrated into the glomerular layer, depending on the age of the cell donor and cell recipient animals. However, consistent with our data, these authors concluded that the fractions of PG cells expressing a set of immunohistochemical markers did not systematically vary for different ages of donor or recipient animals. These data suggest two possibilities as to how biochemical subtype specification is regulated in newly generated neurons. Newly generated neurons may be genetically specified very early on to express different biochemical markers. This idea is supported by the observations that olfactory bulb neurons may be generated from multiple pools of neuronal precursors, one in the SVZ and a second in the RMS which may preferentially generate neurons expressing different markers [4,5]. In another study, genes expressed under the nestin promoter were localized to GLL and not GCL neurons, supporting the idea that there may be different subpopulations of neuronal precursors. This idea that multiple precursor pools exist also was recently supported by a very elegant study in which progeny of specific populations of stem cells were labeled and shown to generate specific subtypes of adult-born neurons [32]. In this case, neurons from different precursor pools containing unique genetic factors may differentiate into diverse subpopulations of olfactory bulb neurons, and preferentially end their migration in different layers.

Conversely, a second explanation is that factors present in the local environment of the olfactory bulb regulate subtype specification. In this case, the reason the population of new neurons resembles the population of mature neurons is because both have been subject to the same cues. Interestingly, the precursor pool for many olfactory bulb neurons born neonatally is different from the pool for adult-born neurons [10], and in rats many of these early proliferating cells survive in the granule cell layer into adulthood. In this way, the distribution of biochemical subtypes of adult-born neurons and that of the existing population would remain similar despite these cells having completely different origins. In addition, the fact that the expression of subtype-specific markers occurs mostly after neurons have arrived in the bulb and migrated to the appropriate layer is consistent with the idea that local regulation might occur. This regulation could take place either at the time of differentiation into various neuronal subtypes via selective survival of certain neuronal subtypes after final differentiation, or via a switch in expression of particular markers. Experiments to determine which of these mechanisms accounts for the regulation of neuronal subtype specification will be critical to understanding the functional role of adult neurogenesis.

## Conclusion

Adult-born neurons in the olfactory bulb differentiate into all of the major subtypes of interneurons examined in this study. This differentiation occurs over a time period from 40–75 days, at the end of which the distribution of subtypes in the population of new born neurons is very similar to the distribution in the population of existing neurons.

## Methods

### Tissue preparation

C57BL/6 mice were given three intraperitoneal (IP) injections of BrdU (60 mg/kg body weight, 15 mg/ml BrdU solution pH 7.2) at two hour intervals and then were then sacrificed at various times after the final BrdU injection (12 hours, 7 days, 14 days, 20 days, 40 days and 75 days). Mice were anesthetized by IP injection of 0.1%/0.1% ketamine/xylazine dissolved in water. Once the animals were non-responsive, they were perfused transcardially with 1% NaCl in 0.1 M phosphate buffer (PB) followed by 4% paraformaldehyde (Sigma) in PB. The brains were extracted and post fixed in 4% paraformaldehyde in PB overnight and then sunk in 30% sucrose in PB. Each hemisphere of the brains was cryogenically sliced into 25 micron sagittal sections using a sliding microtome (Leica SM2000R).

### Double labeling immunofluorescence

Sections from the central 60% of the olfactory bulb (the part containing accessory olfactory bulb) were used for double immunofluorescence labeling. Separate analysis of the medial and lateral most sections revealed no significant differences in the fractions of cells double labeled and so data from all sections were combined for subsequent analysis (fractions of double positive cells in lateral-most, medial-most sections and overall: CR 9 ± 1%, 10 ± 1%, 9 ± 1%; NC 22 ± 5%, 22 ± 3% and 22 ± 2%). Sections from each animal were used for each combination of antibodies, which consisted of anti-BrdU and one of the following: anti-calbindin (CB), anti-calretinin (CR), anti-GABA, anti-N-copine (NC), anti-tyrosine hydroxylase (TH), or anti-NeuN (NeuN). In addition, NeuN was used in combination with anti-S100β (S100B). Sources and dilutions of all antibodies used can be found in Table 1. All incubations were done at room temperature with oscillation. Sections to be labeled with BrdU were first incubated with 2 M HCl for 1 hour to denature the DNA, followed by 2 brief rinses with PB. To permeabilize and block the tissue, the sections were incubated in 0.1% Triton X-100 (Sigma) and 2% normal donkey serum (NDS) (Jackson Immuno Research Laboratories) in PB for 1 hour. The sections were washed 3 times with PB for 10 minutes each wash with oscillation. This wash procedure was also performed following each of the antibody incubations. The antibodies were added in concentrations according to Table 1 in 2% NDS in PB and incubated for 1 hour. The BrdU primary antibody was added followed by the fluorescent BrdU secondary antibody emitting at a red wavelength. Then one of the other primary antibodies was added followed by the corresponding fluorescent secondary antibody emitting at a green wavelength (Table 1). In the case of the combination of S100B and NeuN, the S100B primary antibody was the first in the labeling sequence, and was followed by the fluorescent S100B secondary antibody emitting at a green wavelength, followed by NeuN and its corresponding red secondary antibody. At the addition of the BrdU secondary antibody, all subsequent incubations were performed in the dark. The sections were mounted on collagen coated slides using gelvatol as the mounting medium.

### Neuro Trace labeling

Sections from the central half of the olfactory bulb were co-labeled with the fluorescent Nissl stain NeuroTrace 530/615 red (NTr) or NeuroTrace 500/525 (NTg). (Molecular Probes) and one of BrdU, CB, CR, GABA, NC, or TH. As the only difference between NTr and NTg is the fluorescent color, both stains will be referred to as NT In addition, NT was used to compare NeuroTrace labeling to that of NeuN. or anti-S100B. Four sections from each animal were used. All incubations were done at room temperature with oscillation, and all wash steps consisted of three 10 minute incubations in PB, except where noted. The sections were permeabilized and blocked by incubation in 0.1% Triton X-100 and NDS, and then washed. Tissue to be stained with BrdU was then treated with 25 units/mL of DNAse I (Sigma) in 5 mM MgCl_2 _in PB for 1 hour at 37°C with no oscillation, and then rinsed briefly twice with PB. DNAse was used instead of HCl to denature the DNA because NT staining was not seen in tissue subjected to the HCl wash. In control experiments we observed that while DNAse resulted in less intense staining of BrdU positive cells, similar numbers of cells were BrdU positive in DNAse and HCl treated tissue and the fraction of cells colabeling with secondary markers was similar in the two conditions. All primary and secondary antibody labeling (CB, CR, GABA, NC, TH, NeuN, and S100B) was done as described in the Double Labeling Immunofluorescence section. CB, CR, GABA, N-C, TH, and S100B were labeled with a fluorescent secondary emitting at a green wavelength whereas BrdU and NeuN were labeled with a fluorescent secondary emitting at a red wavelength, (Table 1). Sections were then incubated with 1:100 NTg or NTr, as appropriate, in PB for 20 min, and then washed once with 0.1% TX-100 in PB for 10 min, twice more with PB only for 10 min, followed by a final wash step of PB for two hours. Sections were then mounted on collagen coated slides using gelvatol as the mounting medium.

### Microscopy and image processing

For all sections, 12 bit grayscale images were acquired using an Olympus BX51 microscope fitted with an Optronics Microfire camera and a Ludl x-y-z motorized stage, and mounted on a tabletop vibration isolation table (TMC).

For imaging granule cells stained with CB and CR, two series of images consisting of the entire olfactory bulb were taken with a 10× objective (NA = 0.4). One series visualized the BrdU labeling, and one visualized the CB or CR labeling. These image series were acquired automatically and automatic digital montages were made using the virtual slice module of Neurolucida (Microbrightfield). Exposure times were selected to avoid image saturation. The glomerular region in all virtual slice images was manually masked using Adobe Photoshop, and then the double labeled cells in each image pair were counted using custom software written in Igor Pro (Wavemetrics). Before counting, images underwent background subtraction using a median filtered image, followed by image thresholding to produce a binary image. The threshold value was 5 standard deviations above the mean pixel value of the image. After thresholding, the image underwent contraction and dilation operations to eliminate isolated "noise" pixels. Areas of multiple above-threshold pixels were then identified and their area determined. Groups of connected pixels greater than 9.12 microns (12 pixels) in area were counted as "cells" in single images. This is the approximate area of granule cell nuclei. For counting double positive cells, we combined the binary images taken in the two color channels and created images in which only pixels that were above threshold in both of the single images were above threshold. These images were then counted as above to determine the number of double positive cells. The validity of this process was checked in several control experiments in which the same primary antibody was labeled with two different color secondary antibodies and in other experiments in which two different primary antibodies, directed against the same antigens but made in different species, were used to stain the same tissue. In these experiments our automated double labeling technique was able to identify ~95% of the cells as double positive with a false-positive rate estimated to be less than 2%.

For other antibodies that had higher background staining, and for all glomerular layer imaging, image pairs were acquired using a 40× oil immersion objective (NA = 1.0). For each section, four sets of 40× images of the GCL were acquired, two from the deep GCL and two from the superficial GCL. Four sets of 40× images of the GLL were obtained at even intervals over the GLL. To avoid bias in the selection of regions, we identified cells in the BrdU image, optimized the focus, and then the subsequent CB, CR, GABA, NC, or TH image was obtained without changing the focal plane. Images were combined such that the BrdU images were in the red channel and the appropriate second label (CB, CR, GABA, N-C, or TH) images were in the green channel. These combined images were manually counted by identifying BrdU positive cells, and then overlaying the green channel to determine colocalization of the second label. Cells were identified as double positive only if both the red and green elements were in focus and were much brighter than the background.

For NeuroTrace or NeuN labeled sections, 40× images of the GLL and GCL were acquired as described above. Manual counting of the images was done by visualizing the NT or NeuN channel only; 10 NT or NeuN positive cells were picked at random, and then the co-label channel was overlaid onto the NT or NeuN channel. Cells were identified as double positive as described above.

For all double labeling experiments we took care to avoid counting as double labeled bits of autofluorescent material that appeared frequently but randomly in the glomerular layer. This material, which was also seen in unlabelled tissue, in some cases accounted for a large fraction of the fluorescent objects seen in the glomerular layer, and was very broadly fluorescent, producing bright signals across all filter sets. (See supplemental figure 1). Thus, such spots with strong fluorescence across DAPI, FITC, and Rhodamine filter sets were not counted as being double positive.

In a subset of sections from several animals we compared double counts from epifluorescence images with counts of double labeled cells from confocal images. Immunolabeled sections stained (as above) for CR, N-C and GABA were analyzed using a laser-scanning confocal microscope (LSM 510, Zeiss) and 25× oil objective (Plan-NEOFLAUR, Zeiss). All BrdU labeled cells in a given volume of the granule cell layer were counted by visual inspection with care taken to identify multiple labeled cells in the Z-axis. Cells co-labeled for BrdU and Double labeled cells were subsequently visually identified in the population of BrdU labeled cells. Tissue was analyzed from 4 separate animals for each cellular marker. No significant differences were observed in the fraction of double labeled cells measured by these two different approaches (see Figure 4 and additional file 2).

For all statistical comparisons, we calculated the fraction of double labeled cells across all sections taken from a single animal and we then computed the mean and standard error of these fractions across animals. In all cases significance was computed across groups of animals subject to the same condition.

## Abbreviations

BrdU bromodeoxyuridine

CB calbindin

CR calretinin

EPL external plexiform layer

GABA gamma-aminobutyric acid

GCL granule cell layer

GLL glomerular layer

IP interperitoneal

MCL mitral cell layer

MOB main olfactory bulb

NC N-compine

NeuN neuronal nuclei

NSC neuronal stem cells

NT NeuroTrace

PB phosphate buffer

PG periglomerular

PV parvalbumin

RMS rostral migratory stream

SVZ subventicular zone

TH tyrosine hydroxylase

## Authors' contributions

JB and GML carried out all experiments. NU, JB and GL developed the analysis methods. NU, JB and GL conceived of and designed the experiments. NU, JB and GL wrote and edited the manuscript and prepared the figures. DAJ provided confocal imaging data and edited the manuscript. All authors read and approved the final manuscript.

## Supplementary Material

Additional file 1 Auto-fluorescent particles fluoresce in both color channels in the GLL. Visualizing unlabeled tissue shows the presence of auto-fluorescent particles in the GLL (A). These particles auto-fluoresce under the wavelengths of light used to identify the secondary antibody for (B) BrdU (emits red, excited by 594 nm) and (C) NC, CR, TH, GABA, and CB (emits green, excited by 488 nm), resulting in their appearance as double labeled cells. Scale bars, 74 μm. GLL, glomerular layer; BrdU, bromodeoxyuridine; NC, N-copine; CR, calretinin; TH, tyrosine-hydroxylase; CB, calbindin; MCL, mitral cell layer; EPL, external plexiform layer GCL, granule cell layer. Click here for file

Additional file 2 Confocal imaging taken of sections double labeled for BrdU and GABA (top left) N-copine (bottom left) and calretinin (right). Images show projections and also Z-zxis sections through the cell of interest (indicated by the intersecting lines). In all cases when analyzing double labeling we required that the label in the two colors be overlapping in all three dimensions. Click here for file
