# Supplementary figures and images for: Adult neurogenesis and specific replacement of interneuron subtypes in the mouse main olfactory bulb
Source: BMC Neurosci. 2007 Nov 9;8:92. doi: 10.1186/1471-2202-8-92 (PMC2238759; doi:10.1186/1471-2202-8-92)

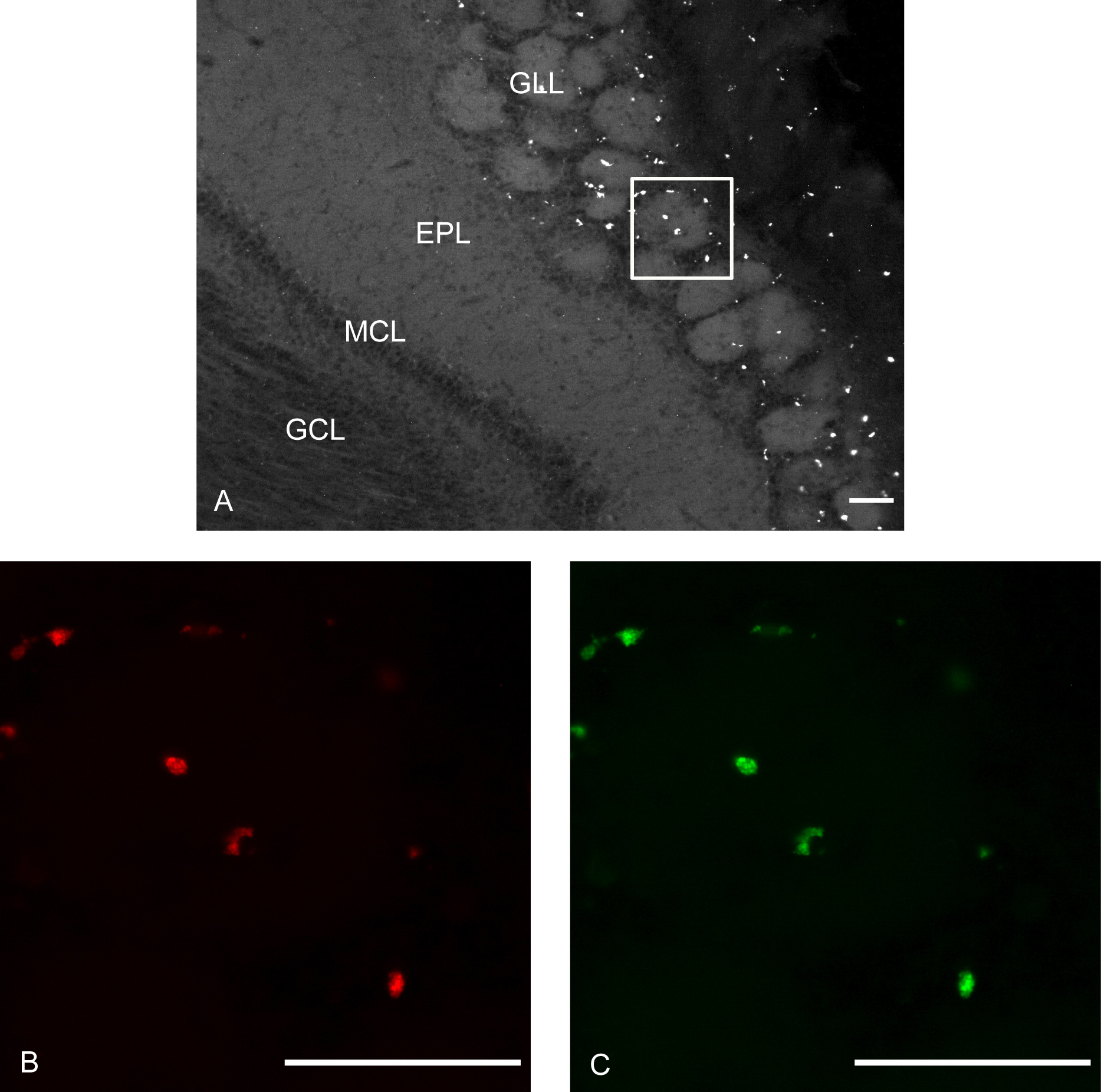

Supplement: Additional file 1 — Auto-fluorescent particles fluoresce in both color channels in the GLL. Visualizing unlabeled tissue shows the presence of auto-fluorescent particles in the GLL (A). These particles auto-fluoresce under the wavelengths of light used to identify the secondary antibody for (B) BrdU (emits red, excited by 594 nm) and (C) NC, CR, TH, GABA, and CB (emits green, excited by 488 nm), resulting in their appearance as double labeled cells. Scale bars, 74 μm. GLL, glomerular layer; BrdU, bromodeoxyuridine; NC, N-copine; CR, calretinin; TH, tyrosine-hydroxylase; CB, calbindin; MCL, mitral cell layer; EPL, external plexiform layer GCL, granule cell layer. [file 1471-2202-8-92-S1.jpeg]

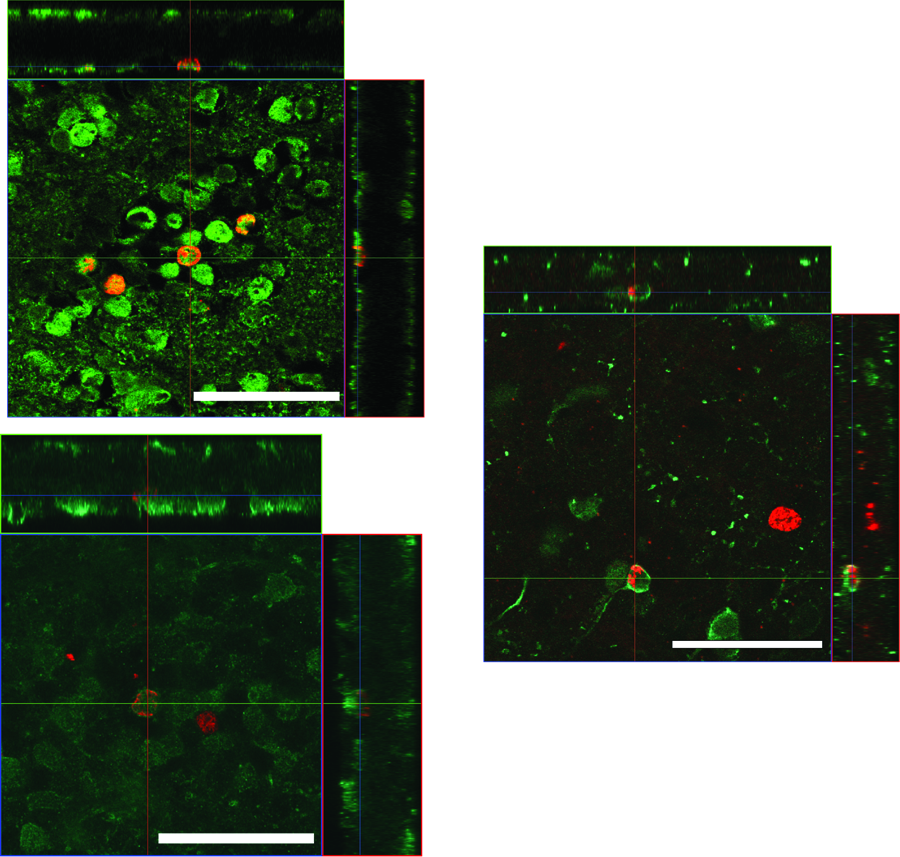

Supplement: Additional file 2 — Confocal imaging taken of sections double labeled for BrdU and GABA (top left) N-copine (bottom left) and calretinin (right). Images show projections and also Z-zxis sections through the cell of interest (indicated by the intersecting lines). In all cases when analyzing double labeling we required that the label in the two colors be overlapping in all three dimensions. [file 1471-2202-8-92-S2.tiff]
